# Evolutionary insights into scleractinian corals using comparative genomic hybridizations

**DOI:** 10.1186/1471-2164-13-501

**Published:** 2012-09-21

**Authors:** Manuel Aranda, Michael K DeSalvo, Till Bayer, Monica Medina, Christian R Voolstra

**Affiliations:** 1 Red Sea Research Center, King Abdullah University of Science and Technology (KAUST), Thuwal, Saudi Arabia; 2 Department of Anesthesia, UCSF School of Medicine, Mission Bay Campus, 600 16th St, Box 2200, San Francisco, CA 94158, USA; 3 School of Natural Sciences, University of California Merced, 5200 North Lake Road, Merced, CA 95343, USA

**Keywords:** Coral reefs, Comparative genomic hybridization (CGH), Microarray, Mitochondria, Evolution

## Abstract

**Background:**

Coral reefs belong to the most ecologically and economically important ecosystems on our planet. Yet, they are under steady decline worldwide due to rising sea surface temperatures, disease, and pollution. Understanding the molecular impact of these stressors on different coral species is imperative in order to predict how coral populations will respond to this continued disturbance. The use of molecular tools such as microarrays has provided deep insight into the molecular stress response of corals. Here, we have performed comparative genomic hybridizations (CGH) with different coral species to an *Acropora palmata* microarray platform containing 13,546 cDNA clones in order to identify potentially rapidly evolving genes and to determine the suitability of existing microarray platforms for use in gene expression studies (via heterologous hybridization).

**Results:**

Our results showed that the current microarray platform for *A. palmata* is able to provide biological relevant information for a wide variety of coral species covering both the complex clade as well the robust clade. Analysis of the fraction of highly diverged genes showed a significantly higher amount of genes without annotation corroborating previous findings that point towards a higher rate of divergence for taxonomically restricted genes. Among the genes with annotation, we found many mitochondrial genes to be highly diverged in *M. faveolata* when compared to *A. palmata*, while the majority of nuclear encoded genes maintained an average divergence rate.

**Conclusions:**

The use of present microarray platforms for transcriptional analyses in different coral species will greatly enhance the understanding of the molecular basis of stress and health and highlight evolutionary differences between scleractinian coral species. On a genomic basis, we show that cDNA arrays can be used to identify patterns of divergence. Mitochondrion-encoded genes seem to have diverged faster than nuclear encoded genes in robust corals. Accordingly, this needs to be taken into account when using mitochondrial markers for scleractinian phylogenies.

## Background

Coral reefs are one of the most productive and diverse ecosystems on our planet. As such, they are of immense ecological and economic importance. Yet, these tropical marine ecosystems are currently threatened by a multitude of factors including climate change-induced mass bleaching events [1], disease [2,3], pollution [4,5], overfishing, and eutrophication [6-8]. Understanding the effects of multiple threats to corals is necessary in order to predict how coral populations will respond to continued disturbance. Genetic and genomic tools now exist that allow us to understand the molecular underpinnings of coral health and stress [9-14].

In particular, cDNA microarrays have accelerated the discovery of stress-responsive genes and mechanisms in recent years in a wide range of non-model organisms [15-17]. cDNA microarrays can assay the expression of thousands of genes simultaneously from control and experimental specimens. Large-scale microarray studies on marine organisms such as porcelain crabs [18], damselfish [19], and gobies [20,21] have provided transcriptomic information in relation to environmental physiology. Small-scale [22,23] and large-scale cDNA microarray studies have been carried out on different scleractinian coral species including *Montastraea faveolata*, *Acropora palmata*, and *Acropora millepora* exposed to environmental stress [9-13,24-27]. However, comparative studies in other coral species are imperative to provide insight into the molecular differences between coral species and to determine the extent to which previous findings can be generalized. Yet, the establishment of new microarray platforms is highly time and resource intensive. Nevertheless, microarray studies are not necessarily restricted to the species from which the cDNAs were generated (i.e. cDNAs from *A. palmata*). Heterologous hybridization is the methodology by which cDNAs from non-reference species are used for hybridization to microarrays (e.g. cDNAs from *Acropora millepora* hybridizing to an *A. palmata* microarray). This process has been described extensively for different non-model organisms including birds, primates, pigs, and bony fish [28-32]. Renn et al. [28] systematically showed that a microarray composed of cDNAs from the African cichlid *Astatotilapia burtoni* yielded biologically meaningful gene expression patterns from heterologous hybridizations spanning evolutionary divergence times from < 10 to > 200 million years (Ma). As expected, the number of spots giving a reliable signal decreased with increasing phylogenetic distance; nevertheless, 3,000–4,000 spots out of 4,500 gave a signal at the largest phylogenetic divergence, which corresponds to 66%–88% of unique spots on the array. Although the ability to detect small fold changes decreases with increasing evolutionary distance, a study on the heat shock response of a damselfish (*Pomacentrus moluccensis*) utilizing an oligonucleotide microarray designed for zebrafish (*Danio rario*-divergence time from 11–300 Ma) reported statistically significant gene expression changes at less than two-fold in magnitude [19].

Prior to hybridizing non-reference cDNAs to a microarray, it is important to use genomic DNA (gDNA) to estimate the projected efficiency of a microarray for heterologous hybridization experiments. The hybridization of gDNA to a cDNA microarray is an example of a comparative genomic hybridization (CGH). In this case gDNA from a non-reference species can be competitively hybridized to the array with gDNA from the reference species, or gDNA from non-reference species can be hybridized alone. The signal intensity of each spot on the microarray is dependent on the sequence similarity and gene copy number between both species (i.e. high sequence divergence = low signal intensity). For example, Renn et al. [28] showed that when labeling gDNA from the reference species *Astatotilapia burtoni*, 93% of spots showed intensity levels two standard deviations over background. In a separate study, gDNA from *Drosophila melanogaster* showed an average of 4.2% greater hybridization than *Drosophila simulans* gDNA to a microarray designed for *D. melanogaster *[33], suggesting that about 95% of the spots yield biological reliable information.

In addition to determining the amount of reliable spots, CGH can also provide valuable information on gene evolution. Numerous studies on *Drosophila *[34], yeast [35,36], *Salmonella *[37], and *Yersinia *[38] have used microarrays to study gene evolution. A particularly relevant study of the ectomycorrhizal fungus *Paxillus involus* and related strains used a cDNA microarray to screen for rapidly evolving genes [39]. Therefore CGH can also be used to identify potentially fast-evolving genes and species-specific adaptations when comparing related species [40].

We have employed CGH against *A. palmata* microarrays containing 13,546 cDNAs using gDNA from *Acropora cervicornis*, *Siderastrea radians*, and *Montastraea faveolata*. This allowed us to: (1) establish the number of “good spots” that can be expected when performing heterologous hybridizations with a range of species at different evolutionary distances; (2) analyze a genome-wide rate of gene evolution; and (3) identify candidates for rapidly diverging genes. Our results show that more than 84% of the spots are likely to provide biologically relevant information across large evolutionary distances (>240 Ma), i.e. the results obtained from these spots can be expected to be scientifically valid. Analyses of the highly divergent gene fractions further provided insights into molecular differences of the two coral clades present today, namely the robust and complex corals, which separated approx. ~240 Ma. Our results suggest that mitochondrial-encoded genes might have played an important role during the evolution of the robust coral clade.

## Results and discussion

### Sequence identity and hybridization signal

**Figure 1:**
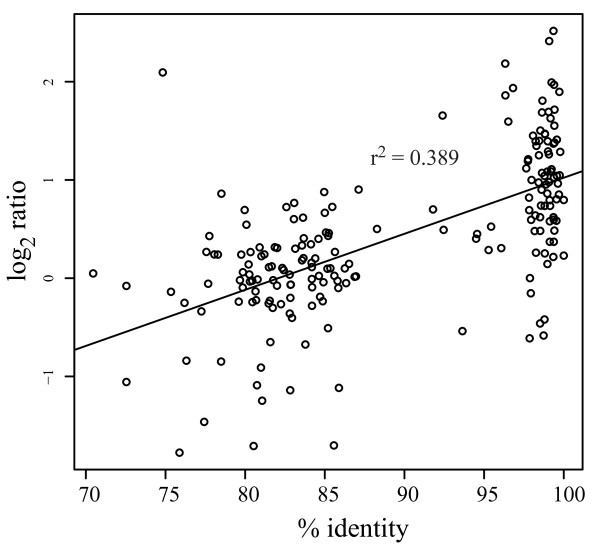
** Linear regression analysis of log**_**2**_**hybridization ratios vs. % sequence identity of *A. palmata* vs.* M. faveolata* orthologs**.

A strong correlation between sequence identity and hybridization signal/ratio is a prerequisite for the use of heterologous hybridizations in interspecies microarray experiments. We used the *A. palmata M. faveolata* comparison to analyze whether hybridization ratios were significantly correlated with the underlying sequence divergence for two reasons: (1) this comparison reflects the largest evolutionary distance in our experiments; and (2) because transcriptome sequence data for *M. faveolata* were readily available. Briefly, orthologs between *A. palmata* and *M. faveolata* were identified by reciprocal tBLASTx of the *A. palmata* spotted cDNAs to a *M. faveolata* transcriptome data set [41]. We compared the 13,546 cDNA clones spotted on the *A. palmata* microarray to 17,703 cDNA sequences from *M. faveolata*. A total of 193 unique spots representing orthologs with alignment lengths above 200 bp were identified and used for subsequent analysis. Linear regression of percent sequence identity (%ID) to log_2_ hybridization ratios of these spots showed a significant correlation (R^2^ = 0.39, p < 0.0001, Figure 1) despite the large evolutionary distance (>240 Ma). Although the correlation observed is not strong, it is similar to what has been observed in previous studies conducted using complete genome sequences in bacteria [42] and *Drosophila* species [34]. These results show that sequence identity and signal intensity are significantly correlated despite a considerable amount of variation and underline the suitability of the *A. palmata* microarray platform for heterologous hybridizations with coral species across large evolutionary distances as has been previously shown for other species [19,28,43]. The variation observed is likely to stem in part from using genomic DNA for the hybridization on cDNA microarray chips. Despite the high identity throughout the coding regions, the spots on the array do not contain any intronic sequences, which might influence the hybridization signal and add to the variation.

### Detection of sequence divergence

In order to determine the amount of suitable spots for heterologous hybridizations with different species, we conducted an Estimated Probability of Presence (EPP) analysis using the software GACK [44]. The EPP analysis assigns a probability for each spotted cDNA sequence of being present (i.e. conserved), slightly divergent, or highly divergent in the non-reference species and therefore allows to statistically identify conserved and divergent genes based on their hybridization signal intensity ratios [44].

**Figure 2:**
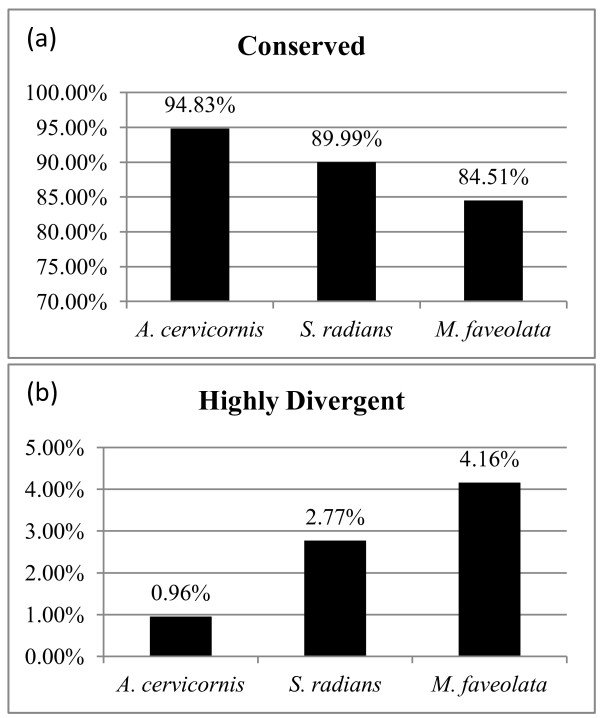
Percentage of (a) conserved and (b) highly divergent genes across species as determined by GACK.

As expected, analysis of the number of divergent genes across species showed an increase of divergent genes and a decrease of conserved genes with increasing evolutionary distance (Figure 2). Specifically, we found that the percentage of conserved genes ranged from 94.83% in the evolutionary closest comparison between *A. palmata* and *A. cervicornis* and 84.51% in the comparison between *A. palmata* and *M. faveolata.* Accordingly we observed an increase of divergent genes from 0.96% to 4.16%. Interestingly, the amount of genes that could not be classified as being either conserved or highly divergent also increased with phylogenetic distance (Figure 2).

**Figure 3:**
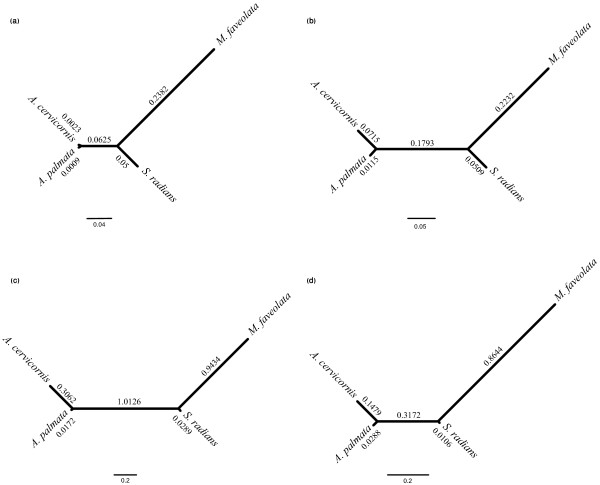
** Unrooted trees of mitochondrial and nuclear genes.** Unrooted Bayesian trees based on (**a**) nucleotide sequence data and (**b**-**d**) hybridization data (genes were assigned either present, slightly divergent, or highly divergent). (**a**) Unrooted tree based on the partial nucleotide sequence of the mitochondrial genes *cox1* and *cytb*. (**b**-**d**) Divergence as inferred from GACK of (**b**) all genes, (**c**) only non-annotated genes, and (**d**) only annotated genes. All trees were generated using Mr. Bayes as described in the methods section.

We used MrBayes [45] to examine the phylogenetic relationships of the coral species using the two mitochondrial genes *cytochrome c oxidase subunit I* (*cox1*) and *cytochrome b* (*cytb*). Sequence data were compared to presence/absence hybridization data as provided by GACK. Both datasets provided trees with identical topology but slight differences in branch lengths indicating that hybridization data recapture sequence-based data and can therefore be used to assess sequence divergence (Figure 3a and b). More specifically, CGH experiments provide a shortcut to assessing sequence divergence in a comparative framework in many different genes and species for a fraction of the cost of sequencing [46]. Interestingly, a comparison of evolutionary trees based on the fraction of annotated and non-annotated genes showed a high increase in branch length separating both acroporids from *S. radians*, which implicates fast divergence of non-annotated genes within the complex corals. A similar increase in branch length is also observed for the complex/robust clade distance; yet, the difference is not as pronounced as with the acroporids and *S. radians* (Figure 3c and d)*.*

We determined the number of unique spots suitable for heterologous hybridization for the different species by defining “good” spots according to their classification in the GACK analysis as being ‘conserved’. Our results showed that more than 94% of the spots are likely to provide biological relevant information for species within the Acroporidae family while we found that >89% of the spots can be used for species of the complex clade and >84% of the spots when using species of the robust clade (Table 1). These percentages represent 12,733, 12,056, and 11,379 spots with respect to the total number of unique spots on the *A. palmata* cDNA platform. The ‘conserved’ gene criterion proved to be a much more conservative approach to determine spot fidelity and resulted in the lowest amount of conserved genes when compared to other methods, such as the use of two standard deviations above background as standard cut-off [28] or methods relying on M values [19,47]. Hence, our approach is likely to underestimate the total amount of suitable spots, especially for more closely related species like *A. cervicornis*. However, we favor a more conservative approach since the correlation of sequence identity and hybridization signal ratios is known to become weaker with increasing sequence divergence [42,47] resulting in impaired biological relevance of data from spots with low hybridization signals. Taken together our data indicate that the *A. palmata* array can be used for heterologous hybridizations with scleractinian coral species from both clades.

**Table 1:** Annoted vs. non-annotated genes

**Genes**	***A. cervicornis***	***S. radians***	***M. faveolata***
**annotated divergent**	44	105	193
**annotated conserved**	6697	5237	4915
**non-annotated divergent**	76	189	255
**non-annotated conserved**	5202	4320	4190
**p-value (chi-square)**	<0.0001	<0.0001	<0.0001

Number of annotated and non-annotated genes in the highly divergent and conserved gene fractions.

### Analysis of divergent and conserved genes

Analysis of the fractions of divergent genes revealed a large number of non-annotated genes across all comparisons. Statistical analysis (Chi square) confirmed a significantly higher number of genes without annotation in the divergent gene fraction across all four species comparisons (p < 0.0001, Table 1). Conversely, annotated genes were significantly overrepresented in the conserved genes fraction (p < 0.0001, Table 1). Comparison of trees generated from either annotated or non-annotated genes showed the same topology, however, the branch lengths were considerably larger for the non-annotated gene fractions (Figure 3), which further shows that non-annotated genes are diverging at a higher rate. Previous studies in *Drosophila,* corals, and *Symbiodinium *[48-50] suggested that non-annotated genes appear to evolve at a higher rate than annotated genes. In general, genes without homologues in other taxa are considered to be lineage- or species-specific and are therefore termed taxonomically restricted genes (TRGs) [51]. TRGs are thought to play an important role in lineage- and species-specific adaptations and have been hypothesized to be a source of phenotypic diversity [52-54]. In scleractinian corals, many genes involved in biomineralization such as some galaxin orthologs appear to be unique to corals and are therefore considered to be coral-specific TRGs [55]. Other TRGs of corals include SCRiPs, a novel family of putatively secreted, small, cysteine-rich proteins that appear to function during development [56].

**Figure 4:**
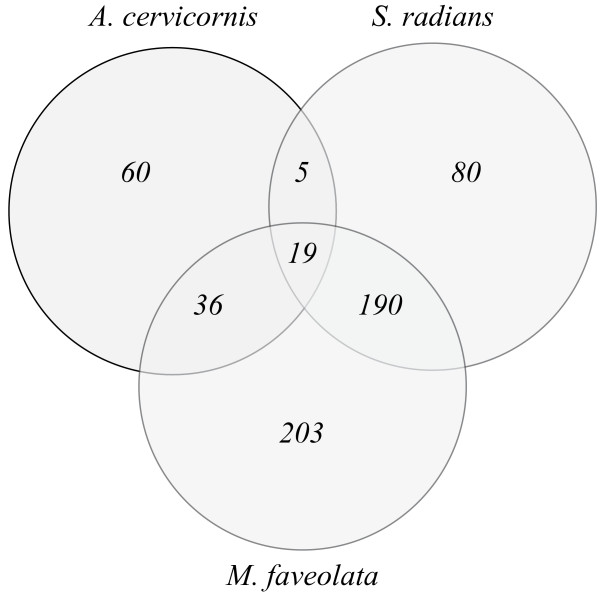
Venn diagram of highly divergent genes shared between species.

We analyzed the overlap of highly divergent genes across all comparisons to identify genes that appear to evolve faster across families and/or clades (Additional file 1). Our analysis showed a successive increase of highly divergent genes with increasing evolutionary distance. We identified a total of 120 unique spots to be highly divergent in *A. cervicornis* and 294 unique spots in *S. radians* when compared to *A. palmata*. Both complex corals shared only 5 unique spots whereas 19 unique spots were shared between all species (Figure 4, Additional file 2). However, it is likely that the 19 unique spots shared between *A. cevicornis*, *S. radians* and *M. faveolata* also contain genes that are actually rapidly diverging in *A. palmata* and hence appear as highly diverged across all species comparisons. The largest overlap of highly divergent genes was found between *S. radians* and *M. faveolata*, which shared 190 unique spots. However, of these 190 unique spots we found 116 to be without annotation and further 37 annotated as predicted, putative, or otherwise uncharacterized protein. A similar result was found for all other comparisons. Of the 60 unique spots found to be highly divergent in the *A. cervicornis* – *A. palmata* comparison only 18 had a functional annotation while only 127 out of the 203 spots unique to *M. faveolata*-*A. palmata* comparison were annotated (Additional file 1, Additional file 3, Additional file 4). The large amount of non-annotated genes in the divergent gene fraction did not allow the identification of specific pathways and/or gene groups that might potentially be rapidly diverging with the exception of mitochondrial genes, which are discussed below.

### Evolution of the robust clade

The comparison between the robust clade (also referred to as the short clade because of their shorter 16 s and 12 s mitochondrial sequences [57,58]) coral *M. faveolata* and the complex coral *A. palmata* revealed 452 putatively divergent genes of which 203 were exclusively divergent in the robust-complex clade comparison, i.e. they did not appear to be divergent in the comparisons within the complex clade corals. Interestingly, these included most of the mitochondrial-encoded genes such as *NADH-ubiquinone oxidoreductase subunits* 1, 4, 5 and 6 as well as *cytochrome c oxidase subunit* 1, 2, 3 and *cytb*. This suggests that the mitochondrial genome of robust corals underwent a phase of rapid divergence while the majority of nuclear encoded genes diverged considerably slower.

Previous studies found that anthozoan mitochondrial genomes display a lower mutation rate than nuclear-encoded genes [59-62]. Hellberg et al. [60] for instance reported that the mitochondrial encoded-gene *cox1* of the two complex corals *Balanophyllia elegans* and *Tubastrea coccinea* showed significantly lower synonymous substitution rates than nuclear-encoded genes. In line with that, Kitahara and colleagues [63] showed that the average nucleotide difference of the mitochondrial *cox1* within the clades was less than 8%. However, the same study showed that the average difference of the *cox1* gene between the complex and the robust clade was 19.1%. Interestingly, phylogenetic comparison between the complex clade and the more basal sister group corallimorpharia showed that the average nucleotide difference of *cox1* was only 13.6%, which is considerably lower than the 21.3% average difference found between robust corals and corallimorpharia. This further suggests that the mitochondrial genome of robust corals must have undergone a phase of rapid divergence during or since the evolutionary split from the complex coral clade.

Indeed, more detailed analysis on the mitochondrial genomes of *Acropora tenuis* and species from the *Montastraea annularis* complex (*M. franksi*, *M. faveolata* and *M. annularis*) showed strong indications for non-neutral and unequal rates of evolution, i.e. the mitochondrial genome of robust corals has been under strong positive selection during or after the evolutionary split of the complex and robust clades [64]. Consequently, Fukami et al. [64] proposed that robust corals might have passed through a general phase of faster evolution. Our results corroborate these findings additionally suggesting that this phase of faster evolution might have been predominantly restricted to the mitochondrial genome while the average divergence rate of nuclear-encoded genes remained largely unchanged. This is an interesting finding which points towards an important role of the coral mitochondrion or mitochondrial-encoded genes during the evolution of the robust clade. For instance, mitochondrial bioenergetics has been discussed as a potential major force in speciation through co-evolution of mitochondrion and nuclear-encoded mitochondrial genes. This can result in specific co-adaptations that can lead to incompatibilities and consequently to reduced fitness and reproductive barriers for certain haplotype combinations [65,66]. Rawson and Burton observed reduced performance for various fitness traits in interpopulation hybrids of the copepod *Tigriopus californicus*, which appeared to be associated with co-adaptation between cytochrome c (nuclear encoded) and cytochrome c oxidase (mitochondrial encoded) [66]. Subsequent analyses suggested a single amino acid substitution in the *cox1* subunit as cause for a lower activity and consequently for the observed interpopulation hybrid breakdown [67].

The evolutionary forces that can lead to co-evolution of nuclear- and mitochondrial-encoded genes are diverse and include climatic adaptation as well as specific adaptations to an ecological niche or changes in the environment [65]. To date it is unclear whether the complex and robust coral clades diverged before or after the Permian-Triassic extinction event [68-71]; yet, both scenarios are in line with strong environmental changes and the sudden availability of new ecological niches. Such strong changes might have favored a rapid adaptation of mitochondrial bioenergetics and thus a phase of rapid divergence of the mitochondrial genome of robust corals.

Corroborating data that the mitochondrial genome underwent a phase of rapid divergence and strong positive selection has interesting implications for current coral molecular phylogenies since many are mainly based on mitochondrial genes [57,58,63,68,70,72]. One of these implications is that the uneven evolutionary rates of coral mitochondrial sequences do not reflect evolutionary divergence time and are therefore suboptimal to resolve phylogenetic relationships within the order Scleractinia. With the complex clade coral genome of *Acropora digitifera* at hand [73] and the robust coral genome of *Stylophora pistillata* being currently sequenced (Voolstra lab at KAUST), we will soon be able to perform phylogenetic analyses using a variety of nuclear-encoded genes that will further shed light on the evolution of the scleractinian coral clades.

## Conclusions

In this study we have demonstrated that the microarray platform available for *A. palmata* can be successfully used to study evolution of scleractinian coral species of both the complex and robust clade. Our results suggest that the platforms currently available might be sufficient to study a wide range of scleractinian coral species, thereby superseding the time and resource consuming development of further platforms for scleractinian coral species. The use of CGH and heterologous hybridizations as tools to (1) study genome-wide gene divergence, (2) identify candidates for rapidly diverging genes, and (3) compare transcriptomic responses to stress among different coral species will greatly enhance our understanding of coral evolution and genomics. While RNAseq might provide higher resolution, microarrays supersede sequencing-based approaches in terms of cost, comparability, and targeted approaches, e.g., compare selected subsets of genes or low expressed genes. Here, we found indications for a potentially important role of the coral mitochondrion/mitochondrial-encoded genes in the evolution of the robust coral clade by analyzing differences in divergence of mitochondrial and nuclear encoded genes. This also has important implications for the use of mitochondrial sequences for scleractinian coral phylogenies.

## Methods

### Coral sampling

Samples of *M. faveolata* and *S. radians* were collected in Puerto Morelos, Mexico during November 2008 on the permit registration MX-HR-010-MEX folio 016. Three colonies of *M. faveolata* were sampled using a hammer and chisel, and three unattached colonies of *S. radians* were taken from a sea grass bed. Three samples of *A. cervicornis* were collected in Bocas del Toro, Panama during March 2008 on the permit SEX/A-26–07–branch tips of three separate colonies were broken off using a hammer and chisel.

### DNA extraction, amplification, and microarray hybridization

Between 50–100 mg of frozen coral tissue were scraped off the samples using a metal corer and DNA was extracted using the PowerPlant DNA extraction kit (MoBio Laboratories, Carlsbad, CA, USA) with the following modifications: following tissue homogenization, samples were spun twice to pellet skeletal debris; and during incubation with Buffer PB1, 1 mg/mL RNase A was added.

Extracted DNA was quantified using a NanoDrop ND-1000 spectrophotometer. Fragmentation of the DNA for whole genome amplification was assessed using the Agilent Bioanalyzer DNA7500 Kit and subsequent fragmentation steps were omitted since the DNA already fulfilled the required fragment size. A total of 25 ng of DNA from each sample was amplified using the GenomePlex Complete Whole Genome Amplification Kit (Sigma Aldrich, Saint Louis, MO, USA) according to the manufacturer’s instructions but using 16 cycles of amplification.

Equal amounts of amplified gDNA from three colonies per species were pooled and subjected to Cy3 and Cy5 labeling using the BioPrime Plus Array CGH Indirect Genomic labeling System (Invitrogen, Carlsbad, CA, USA) in order to account for intraspecific sequence variation. Labeling efficiency was analyzed using a NanoDrop ND-1000 spectrophotometer.

The microarrays used in this study were generated as described in [9] and experiments were performed as follows. Appropriate Cy3 and Cy5 labeled DNAs were mixed together in a hybridization buffer containing 0.25% SDS, 25 mM HEPES and 3 × SSC, resulting in a final volume of 70 μl. The hybridization mixtures were boiled for 2 min at 99°C and allowed to cool at room temperature for 5 min. The cooled hybridization mixtures were pipetted under an mSeries Lifterslip (Erie Scientific), and hybridization took place in Corning hybridization chambers overnight at 55°C. Microarrays were washed once in 2 × SSC, 0.03% SDS heated to 55°C for 5 min. followed by one wash in 1 x SSC and another wash in 0.2 x SSC for 5 min each. The slides were kept in 0.2 × SSC until analysis. Slides were dried via centrifugation and scanned using an Axon 4000B scanner. The experimental setup followed a reference design, i.e., all samples were hybridized against the same pool of labeled *A. palmata* DNA. For each species, a total of four hybridizations were performed, including two dye swap hybridizations in order to account for potential dye bias i.e. two hybridizations with Cy3 labeled *M. faveolata* DNA against a Cy5 labeled *A. palmata* reference and two hybridizations with Cy5 labeled *M. faveolata* DNA against a Cy3 labeled Cy3 *A. palmata* reference were performed. The same hybridization scheme was used for *A. cervicornis* and *S. radians*.

### Data extraction and analysis

Microarray slides were scanned as described in [10]. Spot intensities were extract and background subtracted using TIGR Spotfinder 2.2.3 [74]. The data were quality filtered, and normalized using TIGR MIDAS 2.21 printtip-specific LOWESS [74]. Data have been deposited NCBI’s GEO [75] and are accessible through GEO Series accession number GSE37279. All clone sequences and annotations are available via the EST database: http://sequoia.ucmerced.edu/SymBioSys/index.php.

For all analyses, we only considered spots that were present in at least 3 out of 4 replicates. The log_2_ ratios were averaged per species and the means were used as input for the GACK software [44]. The analysis was performed using the “Trinary Output” option, which classifies genes as either being present (1), slightly divergent (0) or highly divergent (−1). Cut-offs of 10% and 90% probability for present and highly divergent genes were used for subsequent analysis [44].

For the correlation analysis of sequence identity and hybridization signal ratio, the sequences of the probes spotted on the *A. palmata* array were blasted against a *M. faveolata* transcriptome data set and orthologs were determined by using reciprocal tBLASTx [76]. A total of 330 orthologs were identified, of which 193 had alignment lengths >200 bp, and were thus used for subsequent analysis. Plots and statistical analysis were performed using R [77]. Statistical analysis of the distribution of highly divergent and conserved genes across annotated and non-annotated genes was performed with GraphPad Prism 5 using a Chi square test (*df* = 1,*p* < 0.05).

### Phylogeny inference

For phylogenetic analysis of the mitochondrial genes *cox1* and *cytb* we concatenated partial sequences of the following accession numbers. For *cox1*: GenBank:AB441246.1, GenBank:AY451340.1, GenBank:AB441212.1, and GenBank:AF099654.1; for *cytb*: GenBank:AF099655.1, GenBank:AF099654.1, GenBank:DQ643838.1, and GenBank:AF099654.1. Bayesian phylogenetic analysis was performed using MrBayes v3.1.2 [45] using the following settings: nst = 6 for nucleotide data and nst = 1 for divergence data as inferred from GACK, nchains = 4, one cold and three heated chains; the number of steps = generations was set to 2,000,000 with sampfreq = 100 and burnin = 2,500. Convergence was assessed using Tracer v.1.5 [78] and by examining the PSRF values and standard deviation of split frequencies.

## Abbreviations

Ma: Million Years; gDNA: Genomic DNA; CGH: Comparative genomic hybridization; TRGs: Taxonomically restricted genes; SCRiPS: Small cysteine rich proteins.

## Competing interests

The authors declare that they have no competing interests.

## Authors’ contributions

MA wrote the manuscript, designed the microarray study, carried out microarray hybridizations, data extraction and analysis. MKD designed the microarray study, extracted DNA, and wrote the manuscript. TB participated in microarray hybridizations and data analysis. MM participated in study design, and wrote the manuscript. CRV designed the microarray study, participated in microarray hybridizations, data extraction and analysis, coordination, and wrote the manuscript. All authors read and approved the final manuscript.

## Supplementary Material

Additional file 1 Tab delimited txt files showing highly divergent and conserved genes in A. cervicornis. Click here for file

Additional file 2 Tab delimited txt files showing highly divergent genes shared between species. Click here for file

Additional file 3 Tab delimited txt files showing highly divergent and conserved genes in S. radians. Click here for file

Additional file 4 Tab delimited txt files showing highly divergent and conserved genes in M. faveolata. Click here for file

## References

[B1] Hoegh-Guldberg O Climate change, coral bleaching and the future of the world’s coral reefs Marine and Freshwater Research 1999 50 8 839 866 10.1071/MF99078

[B2] Harvell CD Kim K Burkholder JM Colwell RR Epstein PR Grimes DJ Hofmann EE Lipp EK Osterhaus ADME Overstreet RM Emerging marine diseases-climate links and anthropogenic factors Science 1999 285 5433 1505 1510 10.1126/science.285.5433.1505 10498537

[B3] Weil E Smith G Gil-Agudelo DL Status and progress in coral reef disease research Dis Aquat Organ 2006 69 1 1 7 16703761 10.3354/dao069001

[B4] Reopanichkul P Schlacher TA Carter RW Worachananant S Sewage impacts coral reefs at multiple levels of ecological organization Marine Pollution Bulletin 2009 58 9 1356 1362 10.1016/j.marpolbul.2009.04.024 19515390

[B5] Hoegh-Guldberg O Mumby PJ Hooten AJ Steneck RS Greenfield P Gomez E Harvell CD Sale PF Edwards AJ Caldeira K Coral reefs under rapid climate change and ocean acidification Science 2007 318 5857 1737 1742 10.1126/science.1152509 18079392

[B6] Hughes TP Catastrophes, phase shifts, and large-scale degradation of a Caribbean coral reef Science 1994 265 5178 1547 1551 10.1126/science.265.5178.1547 17801530

[B7] Jackson JB Kirby MX Berger WH Bjorndal KA Botsford LW Bourque BJ Bradbury RH Cooke R Erlandson J Estes JA Historical overfishing and the recent collapse of coastal ecosystems Science 2001 293 5530 629 637 10.1126/science.1059199 11474098

[B8] Pandolfi JM Bradbury RH Sala E Hughes TP Bjorndal KA Cooke RG McArdle D McClenachan L Newman MJ Paredes G Global trajectories of the long-term decline of coral reef ecosystems Science 2003 301 5635 955 958 10.1126/science.1085706 12920296

[B9] DeSalvo M Estrada A Sunagawa S Medina M Transcriptomic responses to darkness stress point to common coral bleaching mechanisms Coral Reefs 2012 31 1 215 228 10.1007/s00338-011-0833-4

[B10] Desalvo MK Voolstra CR Sunagawa S Schwarz JA Stillman JH Coffroth MA Szmant AM Medina M Differential gene expression during thermal stress and bleaching in the Caribbean coral Montastraea faveolata Molecular Ecology 2008 17 17 3952 3971 10.1111/j.1365-294X.2008.03879.x 18662230

[B11] DeSalvo MK Sunagawa S Voolstra CR Medina M Transcriptomic responses to heat stress and bleaching in the elkhorn coral Acropora palmata Marine Ecology Progress Series 2010 402 97 113

[B12] Polato NR Voolstra CR Schnetzer J DeSalvo MK Randall CJ Szmant AM Medina M Baums IB Location-specific responses to thermal stress in larvae of the reef-building coral Montastraea faveolata PLoS One 2010 5 6 e11221 10.1371/journal.pone.0011221 20585643 PMC2890407

[B13] Aranda M Banaszak AT Bayer T Luyten JR Medina M Voolstra CR Differential sensitivity of coral larvae to natural levels of ultraviolet radiation during the onset of larval competence Molecular Ecology 2011 20 14 2955 2972 10.1111/j.1365-294X.2011.05153.x 21689186

[B14] Meyer E Aglyamova GV Matz MV Profiling gene expression responses of coral larvae (Acropora millepora) to elevated temperature and settlement inducers using a novel RNA-Seq procedure Molecular Ecology 2011 20 17 3599 3616 21801258 10.1111/j.1365-294X.2011.05205.x

[B15] Gibson G Microarrays in ecology and evolution: a preview Mol Ecol 2002 11 1 17 24 10.1046/j.0962-1083.2001.01425.x 11903901

[B16] Gracey AY Cossins AR Application of microarray technology in environmental and comparative physiology Annu Rev Physiol 2003 65 231 259 10.1146/annurev.physiol.65.092101.142716 12471169

[B17] Hofmann GE Burnaford JL Fielman KT Genomics-fueled approaches to current challenges in marine ecology Trends Ecol Evol 2005 20 6 305 311 10.1016/j.tree.2005.03.006 16701386

[B18] Teranishi KS Stillman JH A cDNA microarray analysis of the response to heat stress in hepatopancreas tissue of the porcelain crab Petrolisthes cinctipes Comp Biochem Physiol Part D Genomics Proteomics 2007 2 1 53 62 10.1016/j.cbd.2006.11.002 20483278

[B19] Kassahn KS Caley MJ Ward AC Connolly AR Stone G Crozier RH Heterologous microarray experiments used to identify the early gene response to heat stress in a coral reef fish Mol Ecol 2007 16 8 1749 1763 10.1111/j.1365-294X.2006.03178.x 17402988

[B20] Buckley BA Gracey AY Somero GN The cellular response to heat stress in the goby Gillichthys mirabilis: a cDNA microarray and protein-level analysis J Exp Biol 2006 209 Pt 14 2660 2677 16809457 10.1242/jeb.02292

[B21] Gracey AY Troll JV Somero GN Hypoxia-induced gene expression profiling in the euryoxic fish Gillichthys mirabilis Proc Natl Acad Sci U S A 2001 98 4 1993 1998 10.1073/pnas.98.4.1993 11172064 PMC29370

[B22] Edge SE Morgan MB Gleason DF Snell TW Development of a coral cDNA array to examine gene expression profiles in Montastraea faveolata exposed to environmental stress Mar Pollut Bull 2005 51 5–7 507 523 16115654 10.1016/j.marpolbul.2005.07.007

[B23] Morgan MB Edge SE Snell TW Profiling differential gene expression of corals along a transect of waters adjacent to the Bermuda municipal dump Marine Pollution Bulletin 2005 51 5–7 524 533 16256144 10.1016/j.marpolbul.2005.09.023

[B24] Rodriguez-Lanetty M Harii S Hoegh-Guldberg OVE Early molecular responses of coral larvae to hyperthermal stress Mol Ecol 2009 18 24 5101 5114 10.1111/j.1365-294X.2009.04419.x 19900172

[B25] Bay LK Ulstrup KE Nielsen HB Jarmer H Goffard N Willis BL Miller DJ Van Oppen MJH Microarray analysis reveals transcriptional plasticity in the reef building coral Acropora millepora Mol Ecol 2009 18 14 3062 3075 10.1111/j.1365-294X.2009.04257.x 19538339

[B26] DeSalvo MK Sunagawa S Fisher PL Voolstra CR Iglesias-Prieto R Medina M Coral host transcriptomic states are correlated with Symbiodinium genotypes Molecular Ecology 2010 19 6 1174 1186 10.1111/j.1365-294X.2010.04534.x 20149089

[B27] Voolstra CR Schwarz JA Schnetzer J Sunagawa S Desalvo MK Szmant AM Coffroth MA Medina M The host transcriptome remains unaltered during the establishment of coral-algal symbioses Mol Ecol 2009 18 9 1823 1833 10.1111/j.1365-294X.2009.04167.x 19317843

[B28] Renn SC Aubin-Horth N Hofmann HA Biologically meaningful expression profiling across species using heterologous hybridization to a cDNA microarray BMC Genomics 2004 5 1 42 10.1186/1471-2164-5-42 15238158 PMC471549

[B29] Castilho PC Buckley BA Somero G Block BA Heterologous hybridization to a complementary DNA microarray reveals the effect of thermal acclimation in the endothermic bluefin tuna (Thunnus orientalis) Mol Ecol 2009 18 10 2092 2102 10.1111/j.1365-294X.2009.04174.x 19389180

[B30] Gilad Y Rifkin SA Bertone P Gerstein M White KP Multi-species microarrays reveal the effect of sequence divergence on gene expression profiles Genome Res 2005 15 5 674 680 10.1101/gr.3335705 15867429 PMC1088295

[B31] Moody D Zou Z McIntyre L Cross-species hybridisation of pig RNA to human nylon microarrays BMC Genomics 2002 3 1 27 10.1186/1471-2164-3-27 12354330 PMC130049

[B32] Degletagne C Keime C Rey B de Dinechin M Forcheron F Chuchana P Jouventin P Gautier C Duchamp C Transcriptome analysis in non-model species: a new method for the analysis of heterologous hybridization on microarrays BMC Genomics 2010 11 1 344 10.1186/1471-2164-11-344 20509979 PMC2901317

[B33] Ranz JM Castillo-Davis CI Meiklejohn CD Hartl DL Sex-dependent gene expression and evolution of the Drosophila transcriptome Science 2003 300 5626 1742 1745 10.1126/science.1085881 12805547

[B34] Renn SC Machado HE Jones A Soneji K Kulathinal RJ Hofmann HA Using comparative genomic hybridization to survey genomic sequence divergence across species: a proof-of-concept from Drosophila BMC Genomics 2010 11 271 10.1186/1471-2164-11-271 20429934 PMC2873954

[B35] Dunham MJ Badrane H Ferea T Adams J Brown PO Rosenzweig F Botstein D Characteristic genome rearrangements in experimental evolution of Saccharomyces cerevisiae Proc Natl Acad Sci U S A 2002 99 25 16144 16149 10.1073/pnas.242624799 12446845 PMC138579

[B36] Edwards-Ingram LC Gent ME Hoyle DC Hayes A Stateva LI Oliver SG Comparative genomic hybridization provides new insights into the molecular taxonomy of the Saccharomyces sensu stricto complex Genome Res 2004 14 6 1043 1051 10.1101/gr.2114704 15173111 PMC419782

[B37] Porwollik S Wong RM McClelland M Evolutionary genomics of Salmonella: gene acquisitions revealed by microarray analysis Proc Natl Acad Sci U S A 2002 99 13 8956 8961 10.1073/pnas.122153699 12072558 PMC124405

[B38] Hinchliffe SJ Isherwood KE Stabler RA Prentice MB Rakin A Nichols RA Oyston PC Hinds J Titball RW Wren BW Application of DNA microarrays to study the evolutionary genomics of Yersinia pestis and Yersinia pseudotuberculosis Genome Res 2003 13 9 2018 2029 10.1101/gr.1507303 12952873 PMC403674

[B39] Le Quere A Eriksen KA Rajashekar B Schutzendubel A Canback B Johansson T Tunlid A Screening for rapidly evolving genes in the ectomycorrhizal fungus Paxillus involutus using cDNA microarrays Mol Ecol 2006 15 2 535 550 16448419 10.1111/j.1365-294X.2005.02796.x

[B40] Forêt S Kassahn K Grasso L Hayward D Iguchi A Ball E Miller D Genomic and microarray approaches to coral reef conservation biology Coral Reefs 2007 26 3 475 486 10.1007/s00338-007-0206-1

[B41] Schwarz J Brokstein P Voolstra C Terry A Manohar C Miller D Szmant A Coffroth M Medina M Coral life history and symbiosis: Functional genomic resources for two reef building Caribbean corals, Acropora palmata and Montastraea faveolata BMC Genomics 2008 9 1 435 10.1186/1471-2164-9-435 PMC2291459 18298846

[B42] Brunelle BW Nicholson TL Stephens RS Microarray-based genomic surveying of gene polymorphisms in Chlamydia trachomatis Genome Biol 2004 5 6 R42 10.1186/gb-2004-5-6-r42 15186493 PMC463075

[B43] von Schalburg K Rise M Cooper G Brown G Gibbs AR Nelson C Davidson W Koop B Fish and chips: various methodologies demonstrate utility of a 16,006-gene salmonid microarray BMC Genomics 2005 6 1 126 10.1186/1471-2164-6-126 16164747 PMC1239916

[B44] Kim CC Joyce EA Chan K Falkow S Improved analytical methods for microarray-based genome-composition analysis Genome Biol 2002 3 11 Research0065 10.1186/gb-2002-3-11-research0065 PMC133449 12429064

[B45] Huelsenbeck J Ronquist F MrBayes: bayesian inference of phylogenetic trees Bioinformatics 2001 17 754 755 10.1093/bioinformatics/17.8.754 11524383

[B46] Malone J Oliver B Microarrays, deep sequencing and the true measure of the transcriptome BMC Biology 2011 9 1 34 10.1186/1741-7007-9-34 21627854 PMC3104486

[B47] Lindroos HL Mira A Repsilber D Vinnere O Naslund K Dehio M Dehio C Andersson SG Characterization of the genome composition of Bartonella koehlerae by microarray comparative genomic hybridization profiling J Bacteriol 2005 187 17 6155 6165 10.1128/JB.187.17.6155-6165.2005 16109957 PMC1196136

[B48] Voolstra CR Sunagawa S Matz MV Bayer T Aranda M Buschiazzo E Desalvo MK Lindquist E Szmant AM Coffroth MA Rapid evolution of coral proteins responsible for interaction with the environment PLoS One 2011 6 5 e20392 10.1371/journal.pone.0020392 21633702 PMC3102110

[B49] Voolstra CR Sunagawa S Schwarz JA Coffroth MA Yellowlees D Leggat W Medina M Evolutionary analysis of orthologous cDNA sequences from cultured and symbiotic dinoflagellate symbionts of reef-building corals (Dinophyceae: Symbiodinium) Comp Biochem Physiol Part D Genomics Proteomics 2009 4 2 67 74 10.1016/j.cbd.2008.11.001 20403741

[B50] Domazet-Loso T Tautz D An evolutionary analysis of orphan genes in Drosophila Genome Res 2003 13 10 2213 2219 10.1101/gr.1311003 14525923 PMC403679

[B51] Wilson GA Bertrand N Patel Y Hughes JB Feil EJ Field D Orphans as taxonomically restricted and ecologically important genes Microbiology 2005 151 Pt 8 2499 2501 16079329 10.1099/mic.0.28146-0

[B52] Kunin V Cases I Enright A de Lorenzo V Ouzounis C Myriads of protein families, and still counting Genome Biol 2003 4 2 401 10.1186/gb-2003-4-2-401 12620116 PMC151299

[B53] Khalturin K Hemmrich G Fraune S Augustin R Bosch TCG More than just orphans: are taxonomically-restricted genes important in evolution? Trends in Genetics 2009 25 9 404 413 10.1016/j.tig.2009.07.006 19716618

[B54] Tautz D Schmid KJ From genes to individuals: developmental genes and the generation of the phenotype Philos Trans R Soc Lond B Biol Sci 1998 353 1366 231 240 10.1098/rstb.1998.0205 9533124 PMC1692214

[B55] Forêt S Knack B Houliston E Momose T Manuel M Quéinnec E Hayward DC Ball EE Miller DJ New tricks with old genes: the genetic bases of novel cnidarian traits Trends in Genetics 2010 26 4 154 158 10.1016/j.tig.2010.01.003 20129693

[B56] Sunagawa S DeSalvo MK Voolstra CR Reyes-Bermudez A Medina M Identification and gene expression analysis of a taxonomically restricted cysteine-rich protein family in reef-building corals PLoS One 2009 4 3 e4865 10.1371/journal.pone.0004865 19283069 PMC2652719

[B57] Romano S Palumbi S Molecular evolution of a portion of the mitochondrial < i > 16S ribosomal gene region in scleractinian corals J Mol Evol 1997 45 4 397 411 10.1007/PL00006245 9321419

[B58] Chen CA Wallace CC Wolstenholme J Analysis of the mitochondrial 12S rRNA gene supports a two-clade hypothesis of the evolutionary history of scleractinian corals Mol Phylogenet Evol 2002 23 2 137 149 10.1016/S1055-7903(02)00008-8 12069546

[B59] Shearer TL Van Oppen MJ Romano SL Worheide G Slow mitochondrial DNA sequence evolution in the Anthozoa (Cnidaria) Mol Ecol 2002 11 12 2475 2487 10.1046/j.1365-294X.2002.01652.x 12453233

[B60] Hellberg ME No variation and low synonymous substitution rates in coral mtDNA despite high nuclear variation BMC Evol Biol 2006 6 24 10.1186/1471-2148-6-24 16542456 PMC1431588

[B61] France SC Hoover LL DNA sequences of the mitochondrial < i > COI gene have low levels of divergence among deep-sea octocorals (Cnidaria: Anthozoa) Hydrobiologia 2002 471 1 149 155 10.1023/A:1016517724749

[B62] Chen IP Tang C-Y Chiou C-Y Hsu J-H Wei N Wallace C Muir P Wu H Chen C Comparative analyses of coding and noncoding DNA regions indicate that < i > acropora (Anthozoa: Scleractina) possesses a similar evolutionary tempo of nuclear vs mitochondrial genomes as in plants Marine Biotechnology 2009 11 1 141 152 10.1007/s10126-008-9129-2 18670809

[B63] Kitahara MV Cairns SD Stolarski J Blair D Miller DJ A comprehensive phylogenetic analysis of the Scleractinia (Cnidaria, Anthozoa) based on mitochondrial CO1 sequence data PLoS One 2010 5 7 e11490 10.1371/journal.pone.0011490 20628613 PMC2900217

[B64] Fukami H Knowlton N Analysis of complete miochondrial DNA sequences of three members of the Montastraea annularis coral species complex (Cnidaria, Anthozoa, Scleractinia) Coral Reefs 2005 24 3 410 417 10.1007/s00338-005-0023-3

[B65] Gershoni M Templeton AR Mishmar D Mitochondrial bioenergetics as a major motive force of speciation BioEssays 2009 31 6 642 650 10.1002/bies.200800139 19408245

[B66] Rawson PD Burton RS Functional coadaptation between cytochrome c and cytochrome c oxidase within allopatric populations of a marine copepod Proceedings of the National Academy of Sciences 2002 99 20 12955 12958 10.1073/pnas.202335899 PMC130567 12271133

[B67] Harrison JS Burton RS Tracing hybrid incompatibilities to single amino acid substitutions Mol Biol Evol 2006 23 3 559 564 16280539 10.1093/molbev/msj058

[B68] Medina M Collins AG Takaoka TL Kuehl JV Boore JL Naked corals: skeleton loss in scleractinia Proceedings of the National Academy of Sciences 2006 103 24 9096 9100 10.1073/pnas.0602444103 PMC1482572 16754865

[B69] Stanley GD The evolution of modern corals and their early history Earth-Science Reviews 2003 60 3–4 195 225

[B70] Romano SL Palumbi SR Evolution of scleractinian corals inferred from molecular systematics Science 1996 271 5249 640 642 10.1126/science.271.5249.640

[B71] Stanley GD Fautin DG The origins of modern corals Science 2001 291 5510 1913 1914 10.1126/science.1056632 11245198

[B72] Fukami H Chen CA Budd AF Collins A Wallace C Chuang YY Chen C Dai CF Iwao K Sheppard C Mitochondrial and nuclear genes suggest that stony corals are monophyletic but most families of stony corals are not (Order Scleractinia, Class Anthozoa, Phylum Cnidaria) PLoS One 2008 3 9 e3222 10.1371/journal.pone.0003222 18795098 PMC2528942

[B73] Shinzato C Shoguchi E Kawashima T Hamada M Hisata K Tanaka M Fujie M Fujiwara M Koyanagi R Ikuta T Using the Acropora digitifera genome to understand coral responses to environmental change Nature 2011 476 7360 320 323 10.1038/nature10249 21785439

[B74] Saeed AI Sharov V White J Li J Liang W Bhagabati N Braisted J Klapa M Currier T Thiagarajan M TM4: a free, open-source system for microarray data management and analysis Biotechniques 2003 34 2 374 378 12613259 10.2144/03342mt01

[B75] Edgar R Domrachev M Lash AE Gene Expression Omnibus: NCBI gene expression and hybridization array data repository Nucleic Acids Res 2002 30 1 207 210 10.1093/nar/30.1.207 11752295 PMC99122

[B76] Telford MJ Phylogenomics Current Biology 2007 17 22 R945 R946 10.1016/j.cub.2007.09.023 18029242

[B77] Team RDC R: A language and environment for statistical computing 2008 R Foundation for Statistical Computing, Vienna

[B78] Drummond A Rambaut A BEAST: Bayesian evolutionary analysis by sampling trees BMC Evolutionary Biology 2007 7 1 214 10.1186/1471-2148-7-214 17996036 PMC2247476

